# Varicella zoster and fever rash surveillance in Lao People’s Democratic Republic

**DOI:** 10.1186/s12879-019-3990-7

**Published:** 2019-05-08

**Authors:** Phonethipsavanh Nouanthong, Judith M. Hübschen, Somxay Billamay, Sodaly Mongkhoune, Keooudomphone Vilivong, Vilaysone Khounvisith, Regina Sinner, Marc Grandadam, Darouny Phonekeo, Antony P. Black, Claude P. Muller

**Affiliations:** 1 Institut Pasteur du Laos, Vientiane, Lao People’s Democratic Republic; 2 Department of Infection and Immunity, Luxembourg Institute of Health, 29, rue Henri Koch, L-4354 Esch-sur-Alzette, Grand Duchy of Luxembourg; 3 Children’s Hospital, Vientiane, Lao People’s Democratic Republic; 4 Arbovirus and Emerging Viral diseases Laboratory, Institut Pasteur du Laos, Vientiane, Lao People’s Democratic Republic; 5 Laboratoire National de Santé, Dudelange, Luxembourg

**Keywords:** Varicella, Fever-rash, Measles, Surveillance, Serostudy

## Abstract

**Background:**

In Lao PDR, the epidemiology of varicella infection is uncertain, since it is not a notifiable disease and VZV outbreaks are rarely reported as fever/rash (F/R) diseases.

**Methods:**

We estimated the seroprevalence of VZV (IgG ELISA) in different age cohorts (9 months to 46 years; *N* = 3139) and investigated VZV and 6 other viruses in patients during F/R outbreaks and in an ad hoc sentinel site in the context of the national reporting system (IgM ELISA, PCR).

**Results:**

At least 80% of the sampled population had evidence of VZV infection before the age of 15. The largest increase in seroprevalence occurred between the age groups 1 to 5 and 6 to 7 year-olds*.* A VZV outbreak (clade 2) also occurred in this age group mostly during the first year of primary school (median age 6 years, interquartile range 4.0–7.5). During a dengue outbreak, 6% had varicella. At our F/R sentinel site, 14% of children with viral etiology were laboratory diagnosed as varicella and among others, a sizeable number of measles (*N* = 12) and rubella cases (*N* = 25) was detected compared to those reported for the whole country (*N* = 56 and 45), highlighting nationwide a large challenge of underreporting or misdiagnosis of these notifiable diseases because of lack of diagnostic laboratory capacity.

**Conclusion:**

We recommend strengthening the clinical and laboratory diagnosis of VZV, measles and rubella, the surveillance and reporting of notifiable F/R diseases by retraining of healthcare workers and by setting up sentinel sites and enhancing laboratory capacity.

**Electronic supplementary material:**

The online version of this article (10.1186/s12879-019-3990-7) contains supplementary material, which is available to authorized users.

## Background

Fever and rash (F/R) are common presentations of viral or bacterial infections in children. Viral infections associated with F/R syndromes include measles, rubella, dengue, chikungunya, parvovirus B19, human herpesvirus 6 (HHV6) and 7 (HHV7), Epstein Barr virus, cytomegalovirus, varicella zoster virus (VZV) and others [1]. The incidence and prevalence of these infections widely vary between countries and regions [1], and are not yet defined in Lao PDR, where laboratory diagnostic capacities for most of these viruses are very limited. Knowledge about disease incidence and prevalence can support clinical diagnosis. In the case of VZV, clinical diagnosis is more reliable than for most of the other viral infections causing F/R.

Although typically a mild disease, primary VZV infection may occasionally also be fatal, especially in neonates, the elderly and immunocompromised individuals [2]. Infection of pregnant women can lead to congenital varicella syndrome or other birth defects in the infants. Furthermore, reactivation of latent infection and herpes zoster can be a serious and painful condition especially in the elderly [3].

Primary VZV infections usually occur during early childhood at least in temperate climates. Some studies suggest that in tropical countries exposure and seroconversion occur less frequently and later in adolescence, leaving a significant proportion of adults, including pregnant women and even elderly susceptible to primary infection [4–8].

Vaccination can significantly reduce varicella-associated disease burden and hospitalization [9, 10]. VZV vaccine is used in many developed countries, however, in developing countries, including Lao PDR, VZV vaccine is not part of the routine childhood vaccination program [11].

Lao PDR is a land-locked country in Southeast Asia with high humidity and high ambient temperatures. No VZV seroprevalence data exist from the general Lao population and the epidemiology of varicella infection is uncertain, since it is not a notifiable disease. Moreover, VZV outbreaks are rarely reported as F/R disease [12]. Here, we estimated the seroprevalence of VZV in different cohorts in the country and investigated VZV in patients with F/R in the context of the Lao reporting system for F/R diseases.

### Participants and methods

All protocols were approved by the Lao National Ethics Committee for Health Research (NECHR). Written informed consent was obtained from adult participants and the parents or legal guardians of children and infants. Additional file 1: Figure S1 shows an overview of the methodology.

### Fever-rash outbreak in northern Lao PDR

The National Centre for Laboratory and Epidemiology (NCLE) was notified of a suspected measles outbreak in a remote region of Pakseng district, Luang Prabang Province in January 2015. The investigation team included provincial health staff, epidemiologists and laboratory scientists from NCLE, WHO, and Institut Pasteur du Laos (IPL). Clinical diagnosis was performed by the local physicians and 29 blood samples were collected from children and 2 from adults who had rash within the previous 28 days. Measles and rubella IgM testing was performed by NCLE as described previously [12]. Samples were further tested at IPL for anti-varicella IgM antibodies (anti-VZV IgM ELISA, Euroimmun). In addition to the sera, throat swabs were collected from 42 cases and investigated by PCR and sequencing as described before [13].

### Seroprevalence of VZV IgG antibodies

Samples from 3139 participants aged 9 months to 46 years, living in different Lao Provinces, were collected between 2011 and 2015. Infants (*n* = 207; mean age 9.4 ± 0.7 months) were recruited when coming for routine measles vaccination to a central hospital in Vientiane and to Luang Prabang provincial hospital in 2011 and 2012 (NECHR ref. 001/2012). Pre-school children (*n* = 803; mean age 2.0 ± 0.9 years) were randomly selected from 26 villages in Kuan and Xamtai districts in Houaphan province in 2013 as described elsewhere [14] (NECHR ref. 732/2013). School children aged between 5 and 19 years (*n* = 1731; mean age 13.1 ± 3.7 years) were recruited in four different provinces (Vientiane Capital, Savannakhet, Bolikhamxay and Luang Prabang) during a measles and rubella (M/R) supplementary immunisation activity in 2011/2012 (NECHR ref. 001/2012). Pregnant women (*n* = 398; mean age 26.3 ± 5.4 years) were recruited from antenatal clinics in Vientiane and Luang Prabang in 2012 (NECHR ref. 001/2012). IgG antibodies were detected using anti-varicella IgG ELISA kits according to manufacturer’s instructions (anti-IgG VZV ELISA, Euroimmun).

### Fever and rash surveillance in children

Between January and December 2015, a total of 511 children presenting with F/R were recruited at the Out-Patients-Department of the Children Hospital, Vientiane, which served here as a sentinel site for active M/R surveillance in the framework of F/R surveillance. Following parental informed consent, throat swabs, oral fluid and blood samples were obtained. IgM antibodies against measles, rubella, parvovirus B19, adenovirus and VZV were measured using Euroimmun IgM ELISA kits.

DNA and RNA were extracted using Nucleospin kits extraction kits (Macherey Nagel). F/R multiplex kits (Fast Track Diagnostics) were used to detect parvovirus B19 and HHV6 and 7 DNA in serum, and measles and enterovirus RNA in throat swabs. Because HHV6 and HHV7 detection in throat swabs is likely to be due to chronic infection and detection in blood is more indicative of acute infection [15, 16], we chose serum for detection of these herpesviruses by PCR.

In participants who were IgM borderline or positive, rubella RNA and VZV DNA were detected from throat swabs and oral fluid, respectively, using published primers [13, 17]. Sequencing was attempted for all of the above samples that were positive by PCR for measles, rubella, parvovirus B19 and VZV. Sequences of open reading frames (ORF) 22 and ORF 62 of the VZV genome were analyzed as previously described [13], using the revised VZV nomenclature [18] . Rubella sequences of the 739 nucleotide region of the E1 gene were analyzed together with reference sequences [19] using MEGA 7 [20].

### Fever-rash etiology in suspected dengue cases

Between March 2012 and September 2013, 108 plasma samples were collected in Vientiane Capital from dengue suspected cases. All these adults and children presented with F/R disease as part of the Lao National dengue surveillance network had no laboratory evidence for an active dengue virus infection. These dengue negative samples were tested for IgM antibodies against measles, rubella, VZV and parvovirus B19 by ELISA (Euroimmun).

### Statistical methods

Statistics were analysed using SPSS package v.21. Chi-squared, independent student t-tests and linear regression were used as appropriate. *p* values less than 0.05 were considered significant.

## Results

### Fever-rash outbreak in northern Lao PDR

A total of 35 sera were collected during a F/R outbreak in Houitone village, Pakseng district, clinically suspected to be measles, were negative for both measles and rubella IgM but 30/34 (88.2%; excluding 1 equivocal) of them were varicella IgM positive. The median age of cases was 6 years (interquartile range, IQR (4.0–7.5)) with 50% females. Clinical signs and epidemiology were compatible with varicella diagnosis [12]. Three of 42 throat swabs (including those from the above 35 patients) were PCR positive and sequencing showed that all viruses in this group of patients belong to clade 2 (Table 1).

**Table 1:** Assignment of VZV sequences to clades based on single nucleotide polymorphisms within ORF 22 and ORF 62 [13]. Only one example per nucleotide pattern and location is shown, the total number is given in brackets behind the strain name; na = not available;? = could not be attributed; European Nucleotide Archive accession numbers: LT984492- LT984535

Strain	ORF 22				ORF 62		Clade
	37,902	38,055	38,081	38,177	107,136	107,165	
VZVs/Houitone.LAO/53.14/V[2] (*n* = 3)	G	C	C	A	T	T	2
VZVs/Vientiane.LAO/3.15/V[5] (*n* = 2)	A	T	C	G	T	T	5
VZVs/Vientiane.LAO/6.15/V[2] (*n* = 14)	G	C	C	na	T	T	2
VZVs/Vientiane.LAO/7.15/V[2]	G	C	C	A	T	T	2
VZVs/Vientiane.LAO/7.15/V (*n* = 2)	A	Y	C	R	T	T	?

### Seroprevalence of VZV IgG antibodies in different age groups and sub-populations

**Fig. 1:**
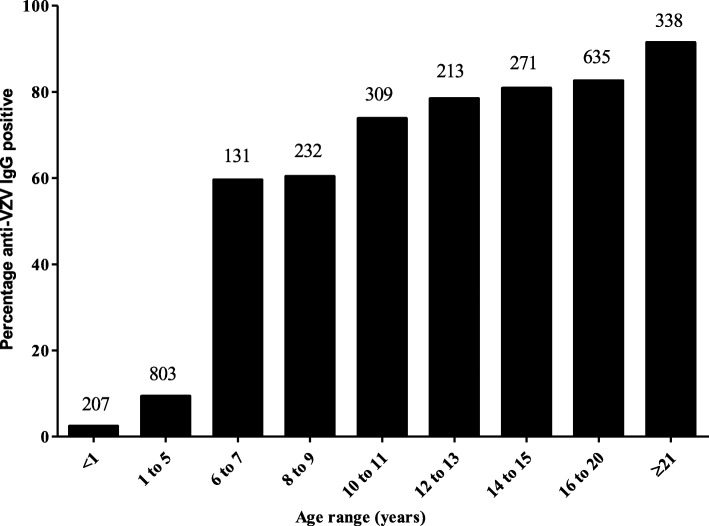
Prevalence of anti-VZV IgG in healthy Lao population by age. Numbers above the bars correspond to total participants per age-group

Anti-VZV IgG was determined in a total of 3139 participants (42.2% male) including 36.9% from urban areas (Vientiane Capital and Luang Prabang) and 63.1% from semi-urban areas (Bolikhamxay, Houaphan and Savannakhet provinces). Overall, 55.6% had IgG antibodies against VZV. The seroprevalence increased with age in both urban and semi-urban areas from 2.4% in infants to 91.5% in women of child bearing age and was significantly correlated with age (r^2^ = 0.94, *p* = 0.017). The largest increase in seroprevalence was between the 1 to 5 age bracket (9.3%) and the 6 to 7 year olds (59.5%; Fig. 1). For all age groups, equivocal samples accounted for between 1.5 and 2.5% and were excluded from the positive results. There were no significant differences between gender, urban and semi-urban areas for any of the cohorts or age groups.

### Children with fever and rash presenting at the children hospital, Vientiane

Following exclusion of 9 participants for whom there was not enough sample for testing and 4 for whom the age was unknown, 498 patients were included in the final analyses. The average age of the children was 2.5 years with a range of 0 to 15.4 years and 45% of the children were female. The majority of F/R cases (88.2%) occurred in children between 0 and 5 years of age. 28.6% (146/511) of participants had confirmed viral etiology (Table 2).

**Table 2:** IgM and viral detection in children with F/R in Vientiane Children Hospital. Numbers indicate the positives among the samples tested. Numbers in brackets are percentages. For HHV6, HHV7 and parvovirus B19 nucleic acid detection, serum samples were used, for measles and enterovirus throat swabs. Numbers include co-detected viruses

Virus	Detection Method	Total (%)	Age (years)			
			0–5	> 5–10	> 10–15	> 15
Parvovirus B19	ELISA (IgM)	4/498 (0.8)	2/440 (0.5)	2/46 (4.3)	0/11	0/1
	PCR	1/498 (0.2)	1/440 (0.2)	0/46	0/11	0/1
Measles	ELISA (IgM)	12/498 (2.4)	10/440 (2.2)	1/46 (2.2)	1/11 (9.1)	0/1
	PCR	2/477 (0.4)	2/421 (0.5)	0/45	0/10	0/1
Rubella	ELISA (IgM)	25/498 (5.0)	22/440 (5.0)	2/46 (4.3)	1/11 (9.1)	0/1
Adenovirus	ELISA (IgM)	39/498 (7.8)	36/440 (8.2)	2/46 (4.3)	1/11 (9.1)	0/1
VZV	ELISA (IgM)	20/498 (4.0)	13/440 (3.0)	5/46 (10.9)	2/11 (18.2)	0/1
Enterovirus	PCR	1/477 (0.2)	1/421 (0.2)	0/45	0/10	0/1
HHV6	PCR	38/498 (7.6)	37/440 (8.4)	1/46 (2.2)	0/11	0/1
HHV7	PCR	11/498 (2.2)	11/440 (2.5)	0/46	0/11	0/1

Adenovirus IgM was the most commonly detected (7.8%), with 17 (43.6%) co-infections or with persisting IgM from a past infection.

VZV IgM was detected in 20 subjects, two thirds of which were below the age of 5 (Table 2). In addition, 10 samples were “equivocal” for VZV IgM. DNA was extracted from oral fluid samples of the 30 participants with positive or equivocal IgM results. Sequencing was attempted for the 19 PCR positive samples. Sequence analyses of the ORFs 22 and 62 showed that 2 viruses belong to clade 5 and 15 to clade 2. Two sequences could not be assigned to specific clades, because of wobbles at important positions (Table 1). Measles IgM was detected in 12 (2.5%) patients, two of whom had positive throat swabs in the diagnostic PCR (Table 2), but not in the sequencing PCR. Rubella IgM was detected in 25 (5.0%) samples, two with PCR-positive throat swabs, one of which was assigned to the genotype 1a vaccine strain. No information about the vaccination status of this patient was available. Parvovirus B19 IgM was detected in 4 (0.8%) samples, with PCR detectable DNA in one of these sera. No sequence was obtained. HHV6 and HHV7 DNA were detected respectively in 38 (7.6%) and 11 (2.2%) sera, all but one in children between 0 and 5 years of age. Only one throat swab was positive for enterovirus by PCR (Table 2). Overall, 20 samples were IgM positive for more than one virus. The number of samples tested was too low to detect any seasonal patterns.

### Fever rash etiology in dengue negative cases

A total of 108 cases with F/R, that were suspected to have dengue, but were not confirmed, were included in this part of the study. The median age of the participants was 20 years with a range of 0 to 60 years. In this cohort IgM antibodies were detected against parvovirus B19 (4; 3.7%), measles (3; 2.8%), rubella (22; 20.4%) and VZV (6/100; 6%). Most IgM positive cases (31/35; 88.6%) were above the age of 10 years.

## Discussion

Our age-stratified seroprevalence findings show that more than 80% of the sample population above the age of 15 had previous varicella infection. In our setting about half of the population seroconverted during the first 2 years of primary school. This is corroborated by the median age of 6 years of the patients in the outbreak in Northern Lao PDR. 90% of the VZV cases in the F/R patients at the Children’s Hospital in Vientiane occurred also before the age of 11. At least 90% of the adult population in this study had been infected. Similarly, an anti-VZV antibody seroprevalence of 95% was recently been reported from a cohort of more than 1100 healthcare workers from three different Lao provinces [21]. With antibodies to VZV in more than 90% of women of child-bearing age, the risk of birth defects and perinatal new-born complications and death is low [2]. This high antibody prevalence is in line with similar observations in other countries in the region [5, 22]. Nevertheless, when vaccination programmes compete for limited resources such as in Lao PDR, VZV vaccination may not be warranted, except for high-risk individuals (e.g. health care workers). Indeed, none of the neighbouring countries in Southeast Asia have introduced VZV into their routine vaccination schedule [23].

The outbreak in Houitane Village, Pakseng District was caused by clade 2 varicella zoster viruses, which were also detected throughout the year 2015, 500 km further south in Vientiane Capital together with at least one other clade (i.e. clade 5). Both clade 2 and clade 5 viruses were also reported from other Asian countries (reviewed in [18]).

Our findings also have some important implications for F/R including M/R surveillance. F/R disease (but not explicitly varicella) is one of 17 diseases reportable to the Lao Early Warning and Response Network (EWARN) surveillance system including the Indicator Based Surveillance (IBS) and Event Based Surveillance (EBS). In general, health facilities do not have the capacity to perform laboratory diagnosis on-site. Therefore, specimens of all suspected F/R cases are supposed to be sent to the National Measles reference laboratory at NCLE for diagnostic confirmation by ELISA and PCR. Testing for other F/R causing pathogens remains challenging in the country.

Nevertheless, few F/R outbreaks are ever notified and brought to the attention of the public health authorities, despite the high incidence of varicella observed in our study, suggesting that disease surveillance and EWARN reporting need to be strengthened.

The outbreak in Northern Lao PDR was initially reported as F/R disease and clinically misdiagnosed as measles, before being confirmed as a varicella outbreak. In the Children’s Hospital, the primary paediatric hospital in the country, about 14% (20/146) of the children with a viral etiology of F/R disease were varicella patients. Also in suspected but unconfirmed dengue cases, 6% were varicella IgM-positive. Thus, overlapping symptoms and a dearth of clinical experience tend to blur the clinical differential diagnosis of varicella and other F/R diseases including M/R in the Lao setting.

In 2015 during the 12 months of our surveillance in the Children’s Hospital in Vientiane, we detected about 500 F/R cases including 12 measles and 25 rubella cases. These are sizeable numbers compared to the 56 measles and 45 rubella cases and the 579 F/R cases reported the same year for the whole country. Also, 3 measles and 22 rubella cases were “hidden” in a cohort of patients (March 2012–September 2013) with F/R suspected to be dengue, compared to 103 and 161 notifications for measles and rubella, in 2012–2013. All of this is evidence of insufficient control of measles (and rubella) despite regular and comprehensive supplementary immunization campaigns at least for measles in addition to the routine outreach vaccinations recommended at 9–24 months of age. It is also an indication of severe underreporting of M/R cases (partially because of weak diagnostic capacity) and a weakness of the F/R surveillance and reporting system.

Among children with F/R about 8% were positive for adenovirus IgM. As adenoviral infections are often subclinical, many of these may not have been the primary reason for seeking medical care [24] but rather represent persisting antibodies or one of the frequent co-infections (43.6%). As in other studies [25, 26], HHV6 and HHV7 infections were common in children under 5 years of age. The low rate of parvovirus B19 infection in the present study is in agreement with its normally mild clinical presentation which does not require medical attention.

In conclusion, we find that varicella has its highest incidence in school children and nearly the whole adult study population has evidence of previous infection. Nevertheless, because of competing programmes, vaccination against varicella may not be indicated for Lao PDR. Our study also warrants several recommendations. (i) Despite considerable efforts of the country to invigorate routine outreach vaccination with large supplementary immunization campaigns, our sentinel surveillance detected a sizeable number of M/R cases that would otherwise have gone unnoticed. Therefore, there is an urgent need to improve M/R surveillance and reporting. (ii) While case based surveillance must continue, even a few sentinel sites could help to monitor surveillance sensitivity to support regional elimination targets. (iii) Including varicella in the list of notifiable diseases may improve F/R and M/R reporting. (iv) Healthcare workers should be retrained in the clinical diagnosis and reporting of F/R diseases in order to increase the sensitivity and specificity of the Lao reporting system and (v) Resources should be made available to ensure that F/R diseases are systematically laboratory investigated at least for M/R and for VZV if clinical diagnosis is unclear.

## Additional file

Additional file 1: **Figure S1.** Over-view of cohorts and methodology used. Flow-chart detailing the methodology of the 4 cohorts used in this study. (PPTX 41 kb)

## Abbreviations

EBS: Event Based Surveillance
EWARN: Lao Early Warning and Response Network
F/R: Fever/rash
HHV: Human herpes virus
IBS: Indicator Based Surveillance
IPL: Institut Pasteur du Laos
Lao PDR: Lao People’s Democratic Republic
M/R: Measles/rubella
NCLE: National centre for laboratory and epidemiology
NECHR: National ethics committee for health research
VZV: Varicella zoster virus
