# Supplementary material for: Varicella zoster and fever rash surveillance in Lao People’s Democratic Republic
Source: BMC Infect Dis. 2019 May 8;19:392. doi: 10.1186/s12879-019-3990-7 (PMC6507166; doi:10.1186/s12879-019-3990-7)
Supplement: Supplementary file 1 — Figure S1. Over-view of cohorts and methodology used. Flow-chart detailing the methodology of the 4 cohorts used in this study. (PPTX 41 kb) [file 12879_2019_3990_MOESM1_ESM.pptx]

## Slide 1
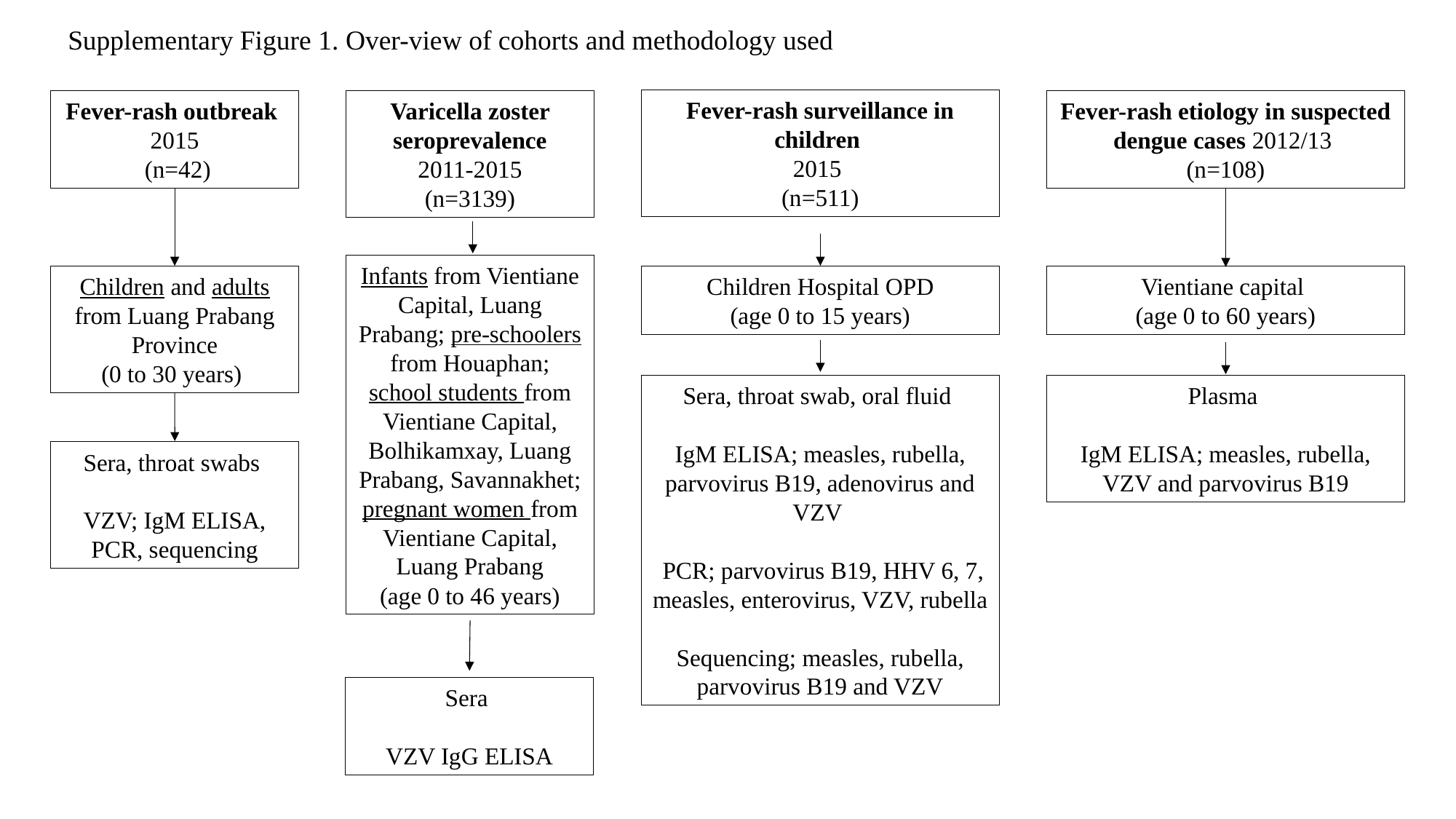

Supplementary Figure 1. Over-view of cohorts and methodology used
Fever-rash surveillance in children
2015
(n=511)
Varicella zoster seroprevalence
2011-2015
(n=3139)
Fever-rash outbreak
2015
 (n=42)
Fever-rash etiology in suspected dengue cases 2012/13
(n=108)
Infants from Vientiane Capital, Luang Prabang; pre-schoolers from Houaphan; school students from Vientiane Capital, Bolhikamxay, Luang Prabang, Savannakhet; pregnant women from Vientiane Capital, Luang Prabang
(age 0 to 46 years)
Vientiane capital
(age 0 to 60 years)
Children and adults from Luang Prabang Province
(0 to 30 years)
Children Hospital OPD
(age 0 to 15 years)
Plasma
IgM ELISA; measles, rubella, VZV and parvovirus B19
Sera, throat swab, oral fluid
IgM ELISA; measles, rubella, parvovirus B19, adenovirus and VZV
 PCR; parvovirus B19, HHV 6, 7, measles, enterovirus, VZV, rubella
Sequencing; measles, rubella, parvovirus B19 and VZV
Sera, throat swabs
VZV; IgM ELISA, PCR, sequencing
Sera
VZV IgG ELISA
